# Outcomes and prognostic factors of endodontically treated teeth filled with calcium silicate‐ or epoxy resin‐based root canal sealers: A retrospective cohort study

**DOI:** 10.1111/iej.14155

**Published:** 2024-10-23

**Authors:** Titanan Kangseng, Danuchit Banomyong, Sittichoke Osiri, Jeeraphat Jantarat

**Affiliations:** ^1^ Department of Operative Dentistry and Endodontics, Faculty of Dentistry Mahidol University Bangkok Thailand; ^2^ Private Practice Bangkok Thailand

**Keywords:** calcium silicate‐based sealer, cold hydraulic condensation, sealer‐based technique, single cone technique, treatment outcome

## Abstract

**Aim:**

The objectives of this study were to compare the clinical outcomes of root canal treatment between calcium silicate‐based sealer using the cold hydraulic condensation technique and epoxy resin‐based sealer using warm vertical compaction and to identify the prognostic factors affecting the outcomes of both obturation techniques.

**Methodology:**

Dental records and radiographs of the teeth that received root canal treatment with calcium silicate‐based sealer using the cold hydraulic condensation technique or epoxy resin‐based sealer using warm vertical compaction during 2015–2021 were selected using inclusion and exclusion criteria. The cases were matched between the two groups based on four factors: primary root canal treatment or retreatment, tooth type, periapical lesion and its size and extension of root canal filling. The success rates of root canal treatment between two sealers/obturation techniques were analysed. The incidence and resorption of extruded sealer were evaluated using the McNemar's test. Generalized estimated equations were used to evaluate any prognostic factors.

**Results:**

The median recall period of this study was 15 months. A total of 234 teeth, comprising 117 cases in each sealer/obturation group, were included. No significant difference was found in the success rates between the calcium silicate‐based sealer using the cold hydraulic condensation technique group (91.5%) and the epoxy resin‐based sealer using the warm vertical compaction group (94.9%). The incidence of sealer extrusion for the calcium silicate‐based sealer (27.6%) did not significantly differ from that of the epoxy resin‐based sealer (36.2%), with both sealers typically demonstrating no resorption at recalls. No prognostic factors related to the success rate of either technique were detected.

**Conclusion:**

The study findings suggest no difference in the success rate between calcium silicate‐based sealer and epoxy resin‐based sealer. The study did not identify any prognostic factors that significantly influenced the outcomes of the endodontic treatments.

## INTRODUCTION

Root canal filling material (i.e. gutta‐percha and sealer) plays an important role in providing the apical seal. Gutta‐percha alone is insufficient for achieving complete root canal sealing. Quality of root canal obturation is a key for endodontic success (Ng et al., 2011a). Warm vertical compaction (WVC) employing heated gutta‐percha and epoxy resin‐based sealer (ERS), such as AH Plus, have emerged as the gold standard in endodontic practice (Jasrasaria et al., 2023; Lee et al., 2017; McMichen et al., 2003). It produces thermoplasticized gutta‐percha which is better flow into root canal space, accessory canals and root canal irregularities. (Camilleri, 2015; Lea et al., 2005). However, this technique required some experience and armamentarium (Iglecias et al., 2017).

In the past decade, calcium silicate‐based sealer (CSS) used with the cold hydraulic condensation (CHC) technique has been developed as supported by substantial research studies (Camilleri et al., 2022; Cardinali & Camilleri, 2023; Donnermeyer et al., 2019). A matched gutta‐percha cone is used as a plunger to create hydraulic pressure for delivering the sealer into the root canal. The technique is effortless and required less equipment (Camilleri et al., 2022).

Calcium silicate‐based sealers are currently available with different compositions and preparations. The sealers are classified into two types according to their preparation form as one‐component and two‐component (Camilleri et al., 2022; Donnermeyer et al., 2019). The one‐component CSS is typically an injectable product in a syringe (premixed or ready‐to‐use) that requires residual moisture inside the root canal and dentinal tubules to initiate the setting reaction. The two‐component CSS is commonly produced in a powder‐and‐liquid or paste‐liquid form that contains water in the liquid component (Camilleri et al., 2022; Cardinali & Camilleri, 2023). Intracanal moisture is not required for the two‐component sealer to set (Badawy & Mohamed, 2022).

There are only a few clinical studies of root canal treatment using CSS/CHC. Two retrospective clinical studies without a control or comparison (Chybowski et al., 2018; Li et al., 2022) reported success (healed) rates of 90.9% and 96.8%. A non‐randomized clinical trial demonstrated similar high success rates between CSS (BioRoot RCS) using CHC (90%) and AH Plus using WVC (89%) at the 1‐year recall (Zavattini et al., 2020). Two other randomized clinical trials also compared the treatment outcomes of CSS and AH Plus (Kim et al., 2022; Song et al., 2022). A very short‐term clinical study compared the outcomes of different CSS (EndoSeal TCS, ADseal, Ceraseal and iRoot SP) to AH Plus at three months. The CSS and AH Plus groups exhibited a nonsignificant difference in the healing rate of pre‐operative periapical radiolucent lesions (33.3% and 38.5%; Song et al., 2022). Another randomized clinical trial compared the success (healed/healing) rates between Endoseal TCS and AH Plus at 17 months and reported non‐significantly different success rates of 94.3% and 92.3% (Kim et al., 2022). Prospective clinical studies compared the success rates (healed) of Ceraseal and AH Plus when filled using the same technique (WVC) at 24 months recall and reported non‐significantly different success rates of 91.1% and 88.6% (Zamparini et al., 2023). Recently, a systematic review and meta‐analysis has shown that there were no differences in tooth survival, treatment outcome, postoperative pain and periapical extrusion between CSS and ERS. The authors suggested that the bulk data had limitations (Zamparini et al., 2024).

Moreover, the major limitation of these previous clinical studies is uncontrolled confounding factors (e.g. tooth type, periapical lesion, or retreatment) between the two obturation groups. A comparison study requires controlling the factors affecting the measured outcomes. To achieve this, a clinical study with a design that matches any confounding factors is required. Therefore, the objective of this matched‐pair, retrospective clinical study was to compare the clinical outcomes of root canal treatment between the CSS/CHC and ERS/WVC obturation techniques and to identify any prognostic factors. The null hypothesis was that there is no difference in the clinical outcomes of root canal treatment between the CSS/CHC and ERS/WVC obturation techniques.

## MATERIALS AND METHODS

This retrospective clinical study was conducted according to the STROBE guideline for observational studies in Endodontics (Vandenbroucke et al., 2014; von Elm et al., 2014). The study protocol was approved by the Institutional Review Board of the Faculty of Dentistry and Faculty of Pharmacy Mahidol University, Bangkok, Thailand (MU‐DT/PY‐IRB 2022/011.2203).

### Sample size calculation

The results of a previous clinical study comparing the two obturation techniques were used to estimate the sample size (Zavattini et al., 2020). The sample size was calculated using nQuery Advisor version 6.01 with the level of significance at 0.05, the power of calculation at 80%, and the difference in proportions between the two groups was 0.1. The calculated sample size was 114 teeth per group, thus at least 228 teeth were recruited.

### Case selection

The dental charts of patients who had teeth that received non‐surgical root canal treatment or retreatment from an endodontic postgraduate student or endodontist in the Endodontic Clinic, Faculty of Dentistry and one Endodontic Department member's private practice between March 2015 and June 2021 were collected following the inclusion/exclusion criteria.

### Inclusion criteria

Endodontically treated teeth in healthy (or well‐controlled systemic disease) patients were included following the inclusion criteria: (i) mature permanent teeth, (ii) root canal filling with gutta‐percha and CSS/CHC, or gutta‐percha and ERS/WVC, (iii) adequate dental records and radiographs and (iv) at least 1‐year recall period.

### Exclusion criteria

The teeth were excluded if one of these conditions was found: (i) major procedural errors in root canal treatment (e.g. separated instrument(s), missed canal(s), or root perforation), (ii) root resorption, (iii) crack or root fracture, (iv) coronal leakage or (v) endo‐periodontal lesion.

### Treatment protocol

The root canal treatments were performed using the multiple‐visit approach using a dental operating microscope by endodontists and postgraduates with standardized protocols for rubber dam isolation, access opening, canal negotiation and working length determination with an electronic apex locator. The canals were then prepared with rotary nickel‐titanium files using the crown‐down technique. The root canals were irrigated with 2.5% sodium hypochlorite (NaOCl) during cleaning and shaping using a 25‐G needle (Nipro, Osaka, Japan), followed by 17% ethylene diamine tetra‐acetic acid (EDTA) as a final irrigant. In some cases, the canals were irrigated with 2% chlorhexidine (CHX) or supplementary irrigated by passive ultrasonic irrigation (PUI) with an Irrisafe tip (Acteon, Ontario, Canada) or XP finisher (FKG Dentaire, La Chaux‐de‐Fonds, Switzerland). Injectable calcium hydroxide paste (Ultracal XS, Ultradent, South Jordan UT, USA), or Triple antibiotic paste were used as an interappointment medication. Cavit G (3 M ESPE, St. Paul, MN, USA) and/or IRM (Dentsply, Caulk, Milford DE, USA) were used to temporarily fill the access cavity.

At the last visit, the patient's signs and symptoms (if any) were resolved before obturation. The intracanal medication was removed, and the root canal was irrigated. The canal was dried using paper points before obturation.

The root canals were filled with gutta‐percha cones and CSS using the CHC technique. The premixed injectable CSS, that is, Ceraseal (Meta Biomed, Cheongju, Korea) and iRoot SP (Innovative Bioceramic Inc., Vancouver, BC, Canada) was filled into the canals by inserting the syringe tip into the coronal to middle portion with or without a lentulo spiral. The matched gutta‐percha master cone(s) was coated with a thin layer of sealer and placed to the working length. The cone was seared off at the orifice level by an electric heat carrier and plugged with an endodontic plugger.

The powder‐liquid CSS was mixed according to the manufacturer's instructions. When the sealer was mixed to the appropriate consistency, a coating of BioRoot RCS (Septodont, Saint‐Maur‐des‐Fosses, France) was applied into the root canal with a gutta‐percha point, and a gutta‐percha master cone (s) was inserted to the working length.

In the case of an oval‐shaped root canal in a premixed injectable CSS and powder‐liquid CSS tooth, accessory cones or a secondary master cone were placed adjacent to the primary master cone without using a spreader.

In the comparison group, the root canals were filled with gutta‐percha cones and AH Plus sealer (Dentsply‐Maillefer, Tulsa, OK, USA) using the WVC filling technique.

Excess sealer was removed and cleaned from the pulp chamber before placing a coronal restoration, which was direct resin composite (with or without a base) as a final or interim restoration before the crown. After completing root canal treatment, the patients were recalled every 6 months.

### Data collection and radiographic evaluation

The clinical data was collected from the chart records and radiographic database. Preoperative, intraoperative and postoperative data were collected. The preoperative data comprised sex, controlled systemic disease, treatment type, single vs. multiple roots, tooth type, pre‐operative periapical lesion, periapical lesion size, pulpal diagnosis, pre‐operative pain, swelling or sinus tract opening, tenderness to percussion, soft tissues tenderness, tooth mobility and probing depth ≥5 mm. The intraoperative data were the irrigant, supplemental irrigation, intracanal medication, procedural error, extension of obturation, sealer extrusion and type of CSS. The postoperative data was the type of coronal restoration.

The digital radiographs were adjusted for brightness and contrast before interpretation. The size of any periapical lesion was measured at its greatest length and width (perpendicular to the length line) using Picture Archiving and Communication system (PACS) version 5.7.100 (FUJIFILM Worldwide, FUJIFILM Medical Systems Inc., Santa Clara, CA, USA). The radiographs were evaluated by two calibrated examiners. The intra‐ and inter‐rater reliability were determined by re‐evaluating 100 radiographs 2 weeks later. In the case of a multi‐root tooth, the worst periapical status of the roots was chosen for evaluation.

### Matched‐pair case selection

The teeth in the CSS/CHC group that met the inclusion criteria were initially recruited. Later, the teeth in the ERS/WVC group (with a higher number of cases due to the longer period of clinical use) were randomly selected and matched following the controlled factors, that is, (i) treatment type (primary treatment/retreatment), (ii) tooth type (anterior, premolar or molar), (iii) presence or absence of a periapical lesion, and lesion size (no, ≤5 mm, >5 mm) and (iv) extension of root canal filling related to the radiographic apex (under‐extension >2 mm, 0–2 mm, over‐extension).

### Outcome assessment

The outcome was assessed from the clinical and radiographic evaluations according to Friedman and Mor's criteria (Friedman & Mor, 2004).

The outcome assessment was initially categorized as:

1. *Healed*: asymptomatic teeth with no apical radiolucency.
2. *Disease*: symptomatic teeth with or without apical radiolucency; or asymptomatic teeth with unchanged, newly emerged, or increased apical radiolucency size.
3. *Healing*: asymptomatic teeth with a decrease in apical radiolucency size

The outcome assessment was finally grouped and dichotomised into: ‘*success*’, which was only the healed, and ‘*failure*’, which was a combination of ‘healing’ and ‘disease’ (Chybowski et al., 2018).

Any sealer extrusion and the persistence of sealer extrusion were recorded.

### Prognostic factors on treatment outcome

The preoperative, intraoperative and postoperative variables were collected to identify any prognostic factors.

### Statistical analysis

The data were analysed using SPSS version 22 for Mac (SPSS Inc., Chicago, IL, USA) and STATA version 17 for Mac (Stata Corp., College Station, TX, USA). The intra‐rater reliability for calibration established with Cohen kappa was 0.87 for the first examiner and 0.85 for the second examiner, and the inter‐rater reliability was 0.89. The kappa score indicated good agreement.

The data distribution, the success rates and the sealer extrusion of the CSS/CHC and AH/WVC groups were compared using the McNemar's test with a significance level of 0.05. The prognostic factors associated with the outcome were initially analysed using the generalized estimating equation (GEE). The factors with a significance level of <0.25 were further included and analysed using a multivariable generalized estimating equation to identify significant outcome predictors.

## RESULTS

### Data distribution

**FIGURE 1:**
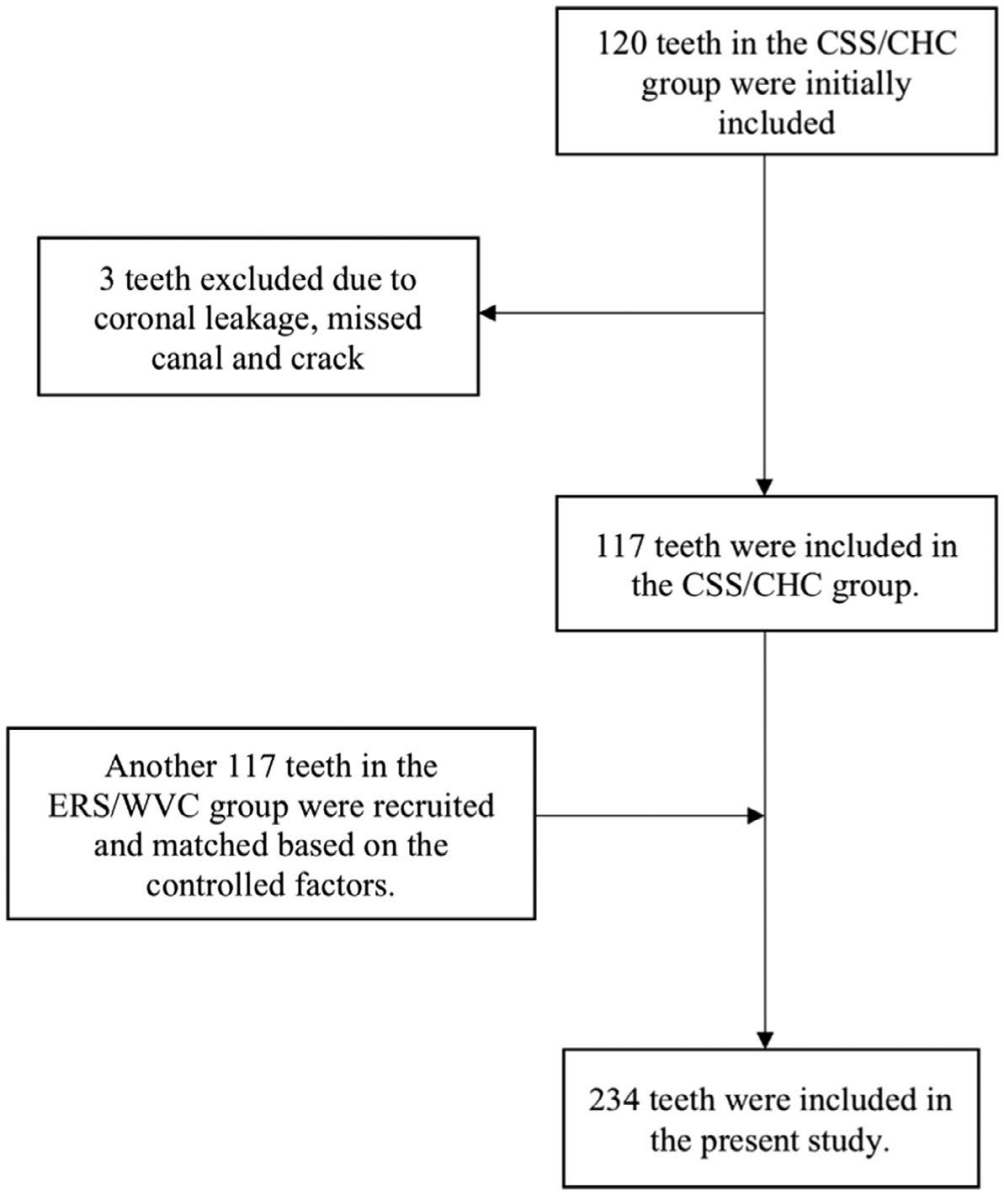
Flow chart illustrating the study flow of teeth fulfilling the inclusion criteria.

In this study, 120 teeth in the CSS/CHC group were initially included. However, three teeth were excluded because of coronal leakage, a missed canal and a crack. Finally, 117 teeth were included in the CSS/CHC group, which were iRoot SP 62 teeth (53%), BioRoot RCS 35 teeth (29.9%) and Ceraseal 20 teeth (17.1%). Another 117 teeth in the ERS/WVC group were later recruited and matched based on the controlled factors (Figure 1).

In each group (*n* = 117), the primary endodontic treatment was 82.9% (97/117). For the percentages of tooth type, molars were 61.5% (72/117), premolars were 20.5% (24/117) and anterior teeth were 18% (21/117). There were 49.6% (58/117) teeth without a pre‐operative periapical lesion, 39.3% (46/117) with a pre‐operative periapical lesion ≤5 mm and 11.1% (13/117) with a pre‐operative periapical lesion >5 mm. Most of the teeth had an obturation length within 0–2 mm from the radiographic apex (113/117), and 3.4% of the teeth (4/117) had short root canal obturation (>2 mm from the apex).

Overall, 234 teeth were recruited from 187 patients comprising 83 males (44.4%) and 104 females (55.6%). The patients were between 12 and 79 years old (average 49.6 ± 15.4 years old).

The data distribution in the two studied groups is presented in Table 1. More than half of the patients were female, 58.1% in the CSS/CHC group and 52.1% in the ERS/WVC group. The percentages of patients with controlled systemic disease(s) were 23.1% in the CSS/CHC group and 27.4% in the ERS/WVC group, with the remaining being healthy. Approximately two‐thirds of the teeth were clinically diagnosed as irreversible pulpitis, that is, 61.9% in the CSS/CHC group and 66% in the ERS/WVC group, respectively. Pre‐operative pain was present in 49 teeth in the CSS/CHC group (41.9%) and 34 teeth in the ERS/WVC group (29.1%). A few teeth in each group had a history of gingival swelling or sinus tract opening, soft tissue tenderness, or tooth mobility. Approximately half of the teeth were tender to percussion, that is, 58.1% in the CSS/CHC group and 44.4% in the ERS/WVC group. Most of the teeth in each group had a normal probing depth (116/117 teeth in both groups). Most of the teeth were medicated with calcium hydroxide for at least 1 week before obturation. Ledges were found in 3 teeth (2.6%) in each group. Sealer extrusion was found in 32 teeth in the CSS/CHC group (27.6%) and 42 teeth in the ERS/WVC group (36.2%). Approximately three‐fourths of the teeth were restored with crowns after endodontic treatment. There was no significant difference in the data distribution between the CSS/CHC and ERS/WVC groups (*p* > .05) except for the irrigant, where the number of teeth irrigated with 2% chlorhexidine CHX in the CSS/CHC group was significantly higher than the ERS/WVC group (*p* < .05). In addition, the number of teeth with supplemental irrigation in the ERS/WVC group was significantly higher than in the CSS/CHC group (*p* < .05).

**TABLE 1:** Data distribution of the teeth in the CSS/CHC and ERS/WVC groups.

Factors	CSS/CHC (*n* = 117)		ERS/WVC (*n* = 117)		*p*‐value
	*n*	%	*n*	%	
Sex					
Male	49	41.9	56	47.9	.419
Female	68	58.1	61	52.1	
Controlled systemic disease					
No	90	76.9	85	72.6	.533
Yes	27	23.1	32	27.4	
Pulpal diagnosis (*n* = 97)					
Irreversible pulpitis	60	61.9	83	66	.541
Necrosis	37	38.1	34	34	
Preoperative pain					
No	68	58.1	105	70.9	.058
Yes	49	41.9	12	29.1	
Swelling or sinus tract opening					
No	108	92.3	65	89.7	.629
Yes	9	7.7	52	10.2	
Tenderness to percussion					
No	49	41.9	112	55.6	.056
Yes	68	58.1	5	44.4	
Soft tissue tenderness					
No	111	94.9	109	95.7	1.000
Yes	6	5.1	8	4.3	
Tooth mobility					
No	112	95.7	116	93.2	.549
Yes	5	4.3	1	6.8	
Probing depth ≥ 5 mm					
No	116	99.1	90	99.1	1.000
Yes	1	0.9	27	0.9	
Irrigant					
NaOCl + EDTA	59	50.4	94	76.9	<.001*
NaOCl + EDTA + Chlorhexidine	58	49.6	23	23.1	
Supplement irrigation					
No	107	91.5	115	80.3	.024*
Yes	10	8.5	2	19.7	
Intracanal medication					
Calcium hydroxide	114	97.4	114	98.3	1.000
Calcium hydroxide + 3‐mix	3	2.6	3	1.7	
Procedural error (ledge)					
No	114	97.4	75	97.4	1.000
Yes	3	2.6	42	2.6	
Sealer extrusion					
No	85	72.4	30	63.8	.184
Yes	32	27.6	87	36.2	
Final restoration					
Direct composite filling	27	23.1	30	25.6	.69
Crown	90	76.9	87	74.4	

*Note*: There was no significant difference in the data distribution of the collected factors between the two groups (the McNemar's test; *p* > .05) except root canal irrigant (*p* < .001), and supplement irrigation (*p* = .024).

* indicates the statistical significance.

### Treatment outcome

The overall median recall period was 15 months (12–72 months). The CSS/CHC group presented 91.5% (107/117 teeth) healed, 6.8% (8/117 teeth) healing and 1.7% (2/117 teeth) disease. The ERS/WVC group exhibited 94.9% (111/117 teeth) healed, 2.6% (3/117 teeth) healing and 2.6% (3/117 teeth) disease (Table 2). There was no significant difference in the success rates according to the strict criteria (healed) between the CSS/CHC and ERS/WVC groups (*p* = .424).

**TABLE 2:** Treatment outcome of the two root canal sealers and obturation techniques.

	Healed *N* (%)	Healing *N* (%)	Disease *N* (%)
CSS/CHC	107 (91.5%)	8 (6.8%)	2 (1.7%)
BioRoot RCS	33 (94.3%)	1 (2.9%)	1 (2.8%)
iRoot SP	57 (91.9%)	5 (8.1%)	0
Ceraseal	17 (85%)	2 (10%)	1 (5%)
ERS/WVC	111 (94.9%)	3 (2.6%)	3 (2.6%)

*Note*: There was no significant difference in the success rates according to the strict criteria (healed) between the CSS/CHC and ERS/WVC groups (*p* = .424). There was no significant difference in the success rates according to the strict criteria (healed) between three different CSS (*p* = .486).

**FIGURE 2:**
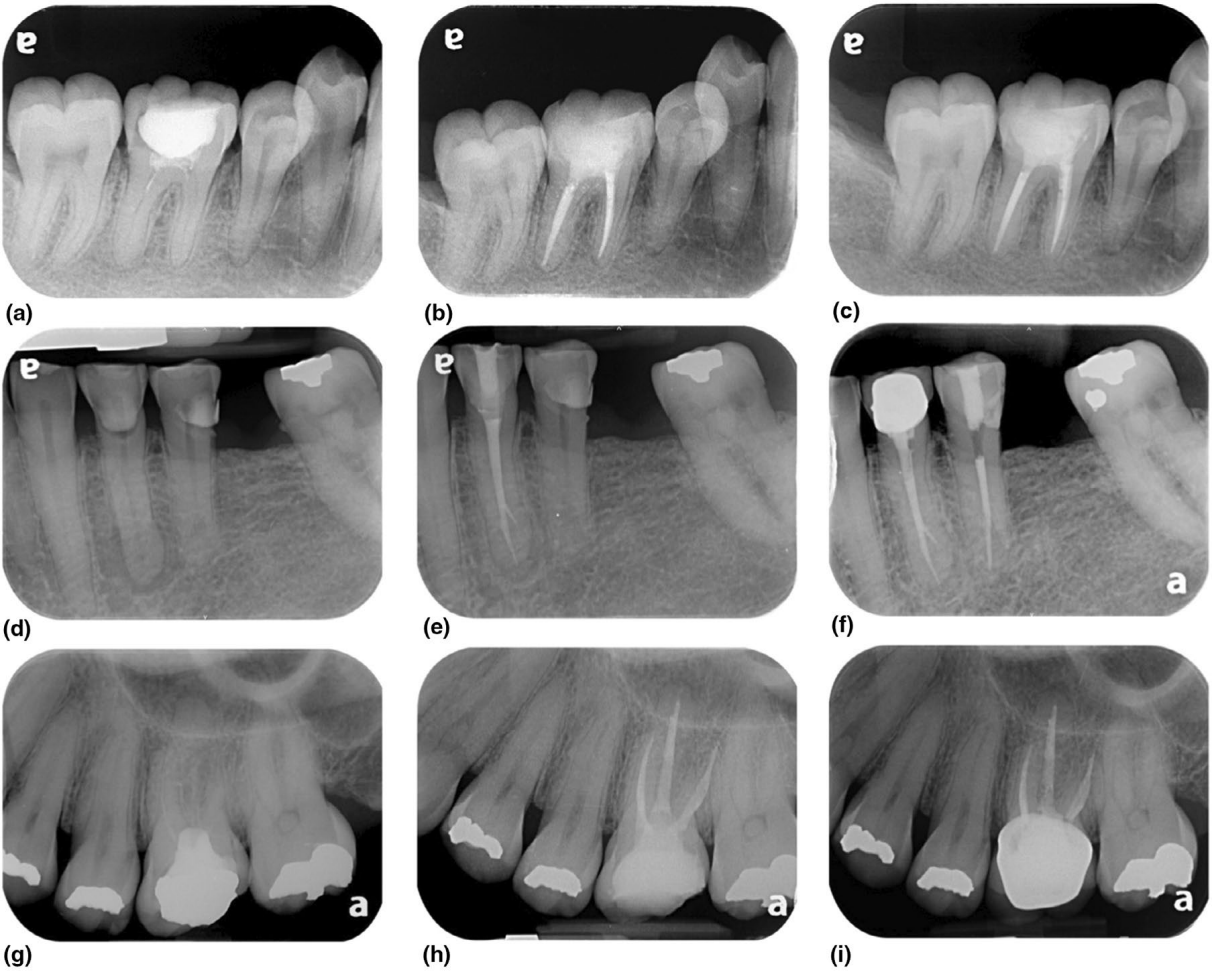
Representative outcome cases. (a–c) Retreatment case of a mandibular molar that healed after a 17‐month recall; (d–f) Mandibular first premolar that healed after a 12‐month recall; (g–i) Maxillary first molar with apical periodontitis after a 12‐month recall. Left column: Preoperative radiographs; Middle column: Postoperative radiographs; Right column: Recall radiographs.

The success rates (healed) of the three different calcium silicate root canal sealers, BioRoot RCS, iRoot SP and Ceraseal were 94.3%, 91.9% and 85%, respectively. The healing rate of BioRoot RCS, iRoot SP and Ceraseal was 2.9%, 8.1% and 10%, respectively. The disease rate of BioRoot RCS and Ceraseal was 2.8% and 5% (Table 2). There was no significant difference in the success rates according to the strict criteria (healed) between three different calcium silicate root canal sealers (*p* = .486; Figure 2).

The success rates (healed) were 91.1% for teeth treated by specialists and 94.4% for those treated by postgraduate students. There was no significant difference in success rates between teeth filled by operators with varying levels of experience (*p* = .326).

### Sealer extrusion

Extruded sealer was found in 32 teeth (27.6%) in the CSS/CHC group and 42 teeth (36.2%) in the ERS/WVC group. There was no significant difference in sealer extrusion between the two groups (*p* = .184). Most of the cases demonstrated extruded sealers; only one tooth (2.4%) in the ERS/WVC group had total resorption of the extruded sealer. Three teeth (7.1%) in the ERS/WVC group and another three teeth (9.4%) in the CSS/CHC group demonstrated partial resorption of extruded sealers. There were two teeth filled with iRoot SP and one tooth filled with BioRoot RCS. The treatment outcome in the case where partial or total sealer resorption occurred was generally healed. However, it is noteworthy that one tooth in the CSS/CHC group exhibited healing.

### Prognostic factors

The bivariate analysis (Table 3) revealed that the teeth with periapical lesions ≤5 mm had a significantly higher success rate (90.2%) than those with periapical lesions >5 mm (73.1%; *p* = .03). A higher success rate was observed in the teeth clinically diagnosed with irreversible pulpitis (96.8%) compared with those with pulp necrosis (88.6%; *p*‐value = .032). Preoperative swelling or sinus tract openings had a significantly negative impact on the root canal treatment outcome (*p* = .03). Tooth mobility also significantly affected the outcome (*p* = .029; Table 3).

**TABLE 3:** Data distribution according to the factors and bivariate analysis for the outcome.

Factors	Number of teeth	Success *n* (%)	Failure *n* (%)	*p*‐value
Sex (*N* = 234)				
Male	105	101 (96.2%)	4 (3.8%)	.109^a^
Female	129	117 (90.7%)	12 (9.3%)	
Controlled systemic disease (*N* = 234)				
No	175	163 (93.1%)	12 (6.9%)	.984
Yes	59	55 (93.2%)	4 (6.8%)	
Treatment type (*N* = 234)				
Primary treatment	194	182 (93.8%)	12 (6.2%)	.389
Retreatment	40	36 (90%)	4 (10%)	
Single versus Multi root (*N* = 234)				
Single root	42	39 (92.9%)	3 (7.1%)	.931
Multi root	192	179 (93.2%)	13 (6.8%)	
Tooth type (*N* = 234)				
Anterior	42	39 (92.9%)	3 (7.1%)	.866
Premolar	48	45 (93.8%)	3 (6.2%)	.965
Molar	144	134 (93.1%)	10 (6.9%)	
Pre‐operative periapical lesion (*N* = 234)				
No	116	116 (100%)	0 (0%)	N/A
Yes	118	102 (86.4%)	16 (13.6%)	
Periapical lesion size (*N* = 118)				
≤5 mm	92	83 (90.2%)	9 (9.8%)	.03^a^
>5 mm	26	19 (73.1%)	7 (26.9%)	
Extension of obturation (*N* = 234)				
Within 0–2 mm	226	210 (92.9%)	16 (7.1%)	N/A
Under extension	8	8 (100%)	0 (0%)	
Pulpal diagnosis (*N* = 194)				
Pulpitis	124	120 (96.8%)	4 (3.2%)	.032^a^
Necrosis	70	62 (88.6%)	8 (11.4%)	
Pain (*N* = 234)				
No	151	139 (92%)	12 (8%)	.369
Yes	83	79 (95.2%)	4 (4.8%)	
Swelling or sinus tract opening (*N* = 234)				
No	213	201 (94.4%)	12 (5.6%)	.03^a^
Yes	21	17 (80.9%)	4 (19.1%)	
Tenderness to percussion (*N* = 234)				
No	114	103 (90.3%)	11 (9.7%)	.106^a^
Yes	120	115 (95.8%)	5 (4.2%)	
Soft tissues tenderness (*N* = 234)				
No	223	207 (92.8%)	16 (7.2%)	N/A
Yes	11	11 (100%)	0 (0%)	
Tooth mobility (*N* = 234)				
No	221	208 (94.1%)	13 (5.9%)	.029^a^
Yes	13	10 (76.9%)	3 (23.1%)	
Probing depth ≥ 5 mm (*N* = 234)				
No	232	216 (93.1%)	16 (7.1%)	N/A
Yes	2	2 (100%)	0 (0%)	
Irrigant (*N* = 234)				
NaOCl + EDTA	149	141 (94.6%)	8 (5.4%)	.244^a^
NaOCl + EDTA + Chlorhexidine	85	77 (90.6%)	8 (9.1%)	
Supplement irrigation (*N* = 234)				
No	201	188 (93.5%)	13 (6.5%)	.582
Yes	33	30 (90.9%)	3 (9.1%)	
Medication w/3‐mix (*N* = 234)				
No	229	213 (93%)	16 (7%)	N/A
Yes	5	5 (100%)	0 (0%)	
Procedural error (ledge) (*N* = 234)				
No	228	212 (93%)	16 (7%)	N/A
Ledge	6	6 (100%)	0 (0%)	
Sealer extrusion (*N* = 234)				
No	160	151 (94.4%)	9 (5.6%)	.285
Yes	74	67 (90.5%)	7 (9.5%)	
Brand sealer (*N* = 117)				
iRoot SP	62	57 (91.9%)	5 (8.1%)	.669
BioRoot	35	33 (94.3%)	2 (5.7%)	.371
Ceraseal	20	17 (85%)	3 (15%)	
Final restoration (*N* = 234)				
Direct composite	57	54 (94.7%)	3 (5.3%)	.59
Crown	177	164 (92.7%)	13 (7.3%)	

*Note*: ‘Success’ was only the ‘healed’, and ‘failure’, which was a combination of ‘healing’ and ‘disease’.

^a^ Factors with *p*‐value < .25 were selected for further analysis using the multivariable generalized estimating equation.

However, the multivariate analysis of the selected factors with *p* < .25 in the bivariate analysis, that is, sex, periapical lesion size, pulpal diagnosis, swelling or sinus tract opening, tenderness to percussion, tooth mobility and irrigant did not identify any significant factor (Table 4).

**TABLE 4:** Multivariable analysis for identifying any prognostic factors to the outcome.

Factors	Odds ratio	95% confidence interval	*p*‐value
Sex			
Male, Female	3.14	0.74–13.40	.12
Periapical lesion size			
≤5 mm, >5 mm	4.43	0.85–23.15	.08
Pulpal diagnosis			
Pulpitis, Necrosis	1.11	0.22–5.73	.90
Swelling or sinus tract opening			
No, Yes	0.58	0.07–4.65	.61
Tenderness to percussion			
No, Yes	0.50	0.11–2.34	.38
Tooth mobility			
No, Yes	5.70	0.78–41.51	.09
Irrigant			
NaOCl + EDTA, NaOCl + EDTA + Chlorhexidine	4.37	0.84–22.64	.08

## DISCUSSION

This retrospective study is the first study concerning the outcome of CSS/CHC that was controlled for the main potential affecting factors, that is, treatment type, tooth type, periapical lesion and extension of root canal filling (Gulabivala & Ng, 2023). Thus, the efficacy of the sealers with cold versus warm filling techniques was compared under similar conditions. Moreover, the study population consisted of 234 teeth, and the sample size calculation was determined to provide statistical power for analysis.

This study compared the teeth filled using different both sealers and obturation techniques, rather than solely focusing on the different materials. The focus was on evaluating the overall performance of obturation techniques that are compatible with each respective sealer (Kooanantkul et al., 2023).

The diversity in clinician experience levels did not exert any discernible influence on the treatment outcomes. Despite the differing levels of experience, the postgraduate students were well‐trained, each possessing at least 1 year of experience in root canal filling techniques with both sealers. Different irrigant protocol and supplement irrigation between the two groups were attributed to the difference in the irrigation protocol in the two study centres. However, the statistical analysis results indicated that these two factors did not exert a significant influence on the outcome.

In retrospective studies, certain information may not be provided. The final apical diameter is influenced by the complexity of each individual case. The type of NiTi used in the study is not specified and varies according to operator preference.

In our study, there was no significant difference in treatment outcomes between the sealers and obturation techniques. Therefore, the null hypothesis was not rejected. Consistent with a previous study, there was no significant difference in the treatment outcome of primary root canal treatment, compared with using BioRoot RCS with the single cone technique and AH Plus with the WVC technique (Zavattini et al., 2020). Similarly, no significant difference was found in the treatment outcome of teeth filled with Endoseal TCS using the sealer‐based technique compared with AH Plus with the WVC technique (Kim et al., 2022).

The absence of a significant difference in treatment outcomes can be attributed to the lack of variance in the density of root canal filling. According to previous clinical research findings, there was no significant difference observed in the presence of voids immediately after canal filling comparing the four different sealers, AH Plus, ADseal, Ceraseal and Endoseal TCS (Song et al., 2022). Another study had similar results, indicating that there was no significant difference in the presence of voids between teeth filled with Endoseal TCS or AH Plus sealer (Kim et al., 2022).

The success of endodontic treatment is accomplished by numerous factors. Root canal treatment aims to achieve debridement, disinfection and obturation of the root canal system within the range of 0–2 mm from the radiographic apex to create a hermetic seal (Byström & Sundqvist, 1981; Sjogren et al., 1990). In addition to the apical seal, the proper coronal seal and cuspal coverage are of paramount importance. In this study, the root canal treatments were performed in multiple‐visits, resulting in the application of medication during each visit as well as using a dental operating microscope by experienced endodontists and postgraduate students with standardized protocols in an academic environment. The previous study compared the outcome of necrotic tooth with apical periodontitis using Bioroot RCS between one‐ and two‐session treatment. The result showed that bioceramic‐based sealers seem to optimize the prognosis of treatment outcome (Bel Haj Salah et al., 2021). Furthermore, another study conducted in a single‐visit setting reported similarly high success rates. The overall success rate of teeth filled with CSS/CHC was 90.9% (Chybowski et al., 2018). Due to the high success rate (>90%), the multivariate analysis did not identify any significant factors.

There were two cases of disease observed in the CSS/CHC group and three cases in the AH/WVC group. Out of these, four cases involved asymptomatic teeth, with two cases showing newly emerged radiographic apical radiolucency and the remaining two cases displaying unchanged radiographic apical radiolucency. In addition, one tooth exhibited a sinus tract opening.

Consistent with previous studies, a higher treatment success rate was observed in cases with smaller periapical lesions (≤5 mm) compared with those with larger lesions (>5 mm). The increased size of these lesions provides a larger area for bacterial colonization and proliferation, leading to a greater bacterial infection burden (Chybowski et al., 2018; Ng et al., 2011a; Pontoriero et al., 2023).

According to a cohort study (Ng et al., 2011b), the success rate of teeth with a pre‐operative sinus tract was lower than teeth without a sinus tract. Teeth with a sinus tract exhibit a complex infectious pattern in the apical root canal system characterized by biofilms that often extend to the outer root surface, leading to the formation of an extraradicular infection. Despite effective disinfection, such as instrumentation, irrigants and medication, the extraradicular infection may persist and cause persistent infection (Ricucci et al., 2018; Siqueira et al., 2014).

The sealer extrusion in both groups was not significantly different. The use of thermoplasticised gutta‐percha as well as the WVC technique is also associated with filling material extrusion (Tennert et al., 2013). However, due to the high flowability of CSS used with an injectable syringe, extruded sealer may occur, particularly in cases where there is a large apical stop or canal over‐preparation. In contrast with other studies, Fonseca et al. reported a greater rate of extrusion for bioceramic sealer compared with AH Plus (Fonseca et al., 2019), Tan et al. found that AH Plus with WVC was more associated with sealer extruded beyond the radiographic apex than TotalFill BC sealer with a sealer‐based technique (Tan et al., 2021). The extruded sealer did not have a significant influence on the treatment outcome, similar to the findings of previous studies (Eriksen et al., 1988; Li et al., 2022; Ørstavik et al., 2004; Ricucci et al., 2016). This can be attributed to the high biocompatibility of the filling material, which promotes the reorganization of the damaged apical tissues that come into contact with the material (Ricucci et al., 2016).

In most of the cases, the extruded sealer was persistent, with only three teeth treated with CSS using the CHC technique and four teeth treated with AH plus using WVC exhibiting resorption of the extruded sealer. This finding could be due to the low solubility of both sealers (Faria‐Júnior et al., 2013; Schäfer & Zandbiglari, 2003), which suggests that they remain stable within the intracanal space (Ricucci et al., 2016).

The laboratory investigations revealed variations in alkalizing activity and other chemical and physical properties amongst the CSSs. Notably, it has been widely demonstrated that BioRoot RCS consistently exhibited higher calcium release values compared with premixed sealers (Bose et al., 2020; Zamparini et al., 2022). With regard to bioactivity (apatite forming ability), Ceraseal nucleates a very thin apatite layer (Zamparini et al., 2022). The powder liquid setting reaction of Bioroot RCS led to a markedly different surface bioactivity reactions compared with premixed sealers (Siboni et al., 2017).

However, this study also compared the outcomes of various CSSs as a result of the operator preference, differentiating between the one‐component form (iRoot SP and Ceraseal) and two‐component form (BioRoot RCS), and found no significant difference in the success rate. Consistent with previous studies, there was no significant difference observed in the treatment outcomes amongst four different CSSs: Ceraseal, BioRoot RCS, AH Plus Bio and BIO‐C SEALER (Pontoriero et al., 2023).

## CONCLUSION

Within the limitations, this retrospective study conducted over a median recall period of 15 months, found no significant difference in the overall success rates between teeth filled with epoxy resin‐based root canal sealer using the warm vertical compaction technique (94.9%) and those filled with calcium silicate‐based root canal sealers using the cold hydraulic condensation technique (91.5%). Amongst the three calcium silicate root canal sealers evaluated, the success rates were 94.3% for BioRoot RCS, 91.9% for iRoot SP, and 85% for Ceraseal. Additionally, the study did not identify any prognostic factors that significantly influenced the outcomes of the endodontic treatments.

## AUTHOR CONTRIBUTIONS

**Titanan Kangseng**: Investigation (lead); Conceptualization (equal); Methodology (equal); Formal Analysis (supportive); writing – original draft (lead). **Danuchit Banomyong**: Conceptualization (equal); Methodology (equal); Investigation (supportive); review and editing (supporting). Supervision (supporting). **Sittichoke Osiri**: Methodology (supporting); Formal Analysis (lead); review and editing (supporting). **Jeeraphat Jantarat**: Conceptualization (equal); Methodology (equal); review and editing (lead). Supervision (lead); Funding Acquisition (lead).

## FUNDING INFORMATION

This research was supported by the Department of Operative Dentistry, Endodontics Division, Faculty of Dentistry, Mahidol University, research fund.

## CONFLICT OF INTEREST STATEMENT

The authors have stated explicitly that there is no conflict of interest in connection with this article.

## ETHICS STATEMENT

The study protocol was approved by the Institutional Review Board of the Faculty of Dentistry and Faculty of Pharmacy, Mahidol University, Bangkok, Thailand (MU‐DT/PY‐IRB 2022/011.2203).

## Data Availability

Data sharing not applicable to this article as no datasets were generated or analysed during the current study.
